# The Essentials of Histology, Descriptive and Practical

**Published:** 1907-12

**Authors:** 


**The Essentials of Histology, Descriptive and Practical.** By **Edward A. Schafer,** F.R.S., Professor of Physiology in University College, London. Seventh edition. Octavo, 507 pages, with 552 illustrations. Lea Brothers and Co., Philadelphia and New York, 1907. (Cloth, $3.50, net).

The student and practitioner will find in this volume, written by the Professor of Physiology of the University of Edinburgh, which is sold for $3.50 and of a size that can be conveniently carried in the pocket, over five hundred pages of the very best matter.

The illustrations are numerous and excellent, the greater portion of them colored, so as to show their actual appearance in stained preparations. The text is clear and refers directly to the cuts which are closely associated with the reading matter.

Lea Bros. & Co., deserve much credit for the perfect work of their printer.

The book is free from laborious and stilted explanation, while its English is clear and without flaw.

The terms employed are those that have been accepted by modern histologists, and we are thankful for the omission of those fanciful and far fetched descriptive terms that only serve to burden the memory and confound the understanding.

The latest knowledge upon the various topics is comprised in the fifty studies that make up the volume and the recent discoveries are all included. That portion treating upon the nervous system contains all that is new and useful with the clearest of explanatory text.

The book contains, so concisely is it written, much more information than many volumes of three times its size and price and will be welcomed by the student as well as by the practitioner who wishes to be abreast of the times. It will be some time before a textbook or work of reference upon this subject will equal, much more excel it.

